# Perceiving humanness across ages: neural correlates and behavioral patterns

**DOI:** 10.3389/fpsyg.2024.1361588

**Published:** 2024-04-04

**Authors:** Toshiki Saito, Rui Nouchi, Ryo Ishibashi, Kosuke Motoki, Yutaka Matsuzaki, Akiko Kobayashi, Motoaki Sugiura, Ryuta Kawashima

**Affiliations:** ^1^Institute of Development, Aging, and Cancer, Tohoku University, Sendai, Japan; ^2^Faculty of Science and Engineering, Waseda University, Tokyo, Japan; ^3^Japan Society for the Promotion of Science, Tokyo, Japan; ^4^Smart Ageing Research Center, Tohoku University, Sendai, Japan; ^5^National Institute of Information and Communications Technology, Osaka, Japan; ^6^Department of Management, The University of Tokyo, Tokyo, Japan; ^7^School of Economics and Management, Kochi University of Technology, Kochi, Japan; ^8^International Research Institute of Disaster Science, Tohoku University, Sendai, Japan

**Keywords:** humanness perception, fMRI, functional connectivity, older adults, dehumanization

## Abstract

Humanness perception, which attributes fundamental and unique human characteristics to other objects or people, has significant consequences for people’s interactions. Notably, the failure to perceive humanness in older adults can lead to prejudice. This study investigates the effect of a target’s age on humanness perception in terms of two dimensions: agency (the ability to act and do) and experience (the ability to feel and sense). We also examined brain activity using a magnetic resonance imaging (MRI) scanner in order to understand the underlying neural mechanisms. Healthy university students viewed the facial images of older and younger individuals and judged the humanness of each individual in terms of agency and experience while inside the MRI scanner. The results indicated that older adults were rated higher on experience, and no difference was found in ratings for agency between younger and older face images. Analysis of brain imaging data indicated that positive functional connectivity between the ventral and dorsal regions of the medial prefrontal cortex (mPFC) was greater when judging the humanness of younger faces than older faces. We also found that the negative functional connectivity between the left inferior frontal gyrus and postcentral gyrus was greater when judging the humanness of older faces as compared to that of younger faces. Although the current study did not show distinct brain activities related to humanness perception, it suggests the possibility that different brain connectivities are related to humanness perception regarding targets belonging to different age groups.

## 1 Introduction

As human beings, the extent to which we attribute humanness or mind toward others varies depending on characteristics such as gender and age. Previous studies have reported that target characteristics influence the perception of humanness, which encompasses sophisticated and experiential capacities distinguishing humans from animals or objects (for a review, see Haslam and Stratemeyer, 2016). For instance, it has been shown that heavier individuals (Sim et al., 2022) and sexualized women (Rudman and Mescher, 2012) tend to be attributed with lower levels of humanness. The extent to which we ascribe humanness to others directs interpersonal activity (Haslam, 2006; Gray et al., 2007). For example, perceiving individuals as being fully human with sophisticated mental faculties leads a perceiver to include them in a moral community or to empathize with them (Haslam and Loughnan, 2014). However, failing to attribute humanness to other people is related to social problems such as prejudice (Drury et al., 2017) and abuse proclivity of caregivers (Chang et al., 2023). Thus, it is important to understand the characteristics that influence the attribution of humanness to others and how humanness is ascribed to others. In addition to body weight and gender, age is an essential aspect in the classification of others (Berry and McArthur, 1986). However, it is unknown whether age that is inferred from facial images influences humanness perception. Therefore, this study addresses this issue.

Convergent evidence suggests that people perceive humanness along two dimensions—agency and experience (Li et al., 2014)—and that the age of the targets may influence both dimensions. Agency refers to the ability to plan and act, and it encompasses self-control, morality, and reasoning, whereas experience refers to the ability to sense and feel emotions and basic psychological states such as hunger and pain (Gray et al., 2007, 2011; Waytz et al., 2010). These dimensions of humanness perception are linked with two dimensions of the stereotype content model (Fiske et al., 2002): warmth and competence. Warmth refers to sociality and morality, thus relating to experience, while competence refers to competitiveness and intelligence, thus relating to agency (Li et al., 2014). Owing to the stereotypical images of older adults, they are perceived as having lower agency and higher experience than younger adults (Waytz et al., 2010). In fact, a larger number of older people struggle with cognitive decline, memory difficulty, or reasoning failure than younger people (Branch et al., 2005), which contributes to their stereotypical images of having lower agency. However, older people are more likely to be perceived as warm and tolerant than younger people (Fiske et al., 2002; Cuddy et al., 2005). Warmth and tolerance are traits associated with the ability to sense and empathize with the emotions of oneself and others. Consequently, for older adults, the stereotypes of being warm and tolerant may contribute to their stereotypical images of having a higher amount of experience. Therefore, we propose the first hypothesis regarding humanness perception toward different age groups:

Participants are likely to perceive the agency of older targets as being lower than that of younger targets, whereas the experience of older targets is likely to be perceived as being higher than that of younger targets.

Previous neuroimaging studies have indicated that specific brain regions—the ventral part of the medial prefrontal cortex (mPFC) and the left inferior frontal gyrus (IFG)—are responsible for humanness perception. Harris and Fiske (2006, 2007) found that mPFC activation, especially in the ventral region, was significantly weaker when participants viewed groups that are perceived as being disgusting—such as homeless people and drug addicts, for example—over non-disgusting groups. They concluded that mPFC activation reflected the tendency to not consider the mind of disgusting groups at the neural level. Another study reported that left-lateralized activity, especially in the left IFG, plays a significant role in dehumanization (Bruneau et al., 2018). In a previous study, participants provided dehumanization ratings to social groups (Americans, Muslims, and the homeless) within a magnetic resonance imaging (MRI) scanner and showed significant activation of the left IFG, even after controlling for other similar ratings, namely dislike and dissimilarity (Bruneau et al., 2018). The researchers interpreted this result as indicating that the left IFG was serving to reduce mentalization toward the targets, thus dehumanizing them by denying their mind. Based on these findings, we propose a second hypothesis regarding the brain regions that are associated with humanness perception:

The ventral mPFC and left IFG are affected by the age of the target during humanness perception. In particular, activation of the ventral mPFC, which can positively correlate with humanness perception, is weaker when perceiving older targets than younger targets because older targets are perceived as having lower agency. However, the activation of the IFG, which can negatively correlate with humanness perception, is weaker when perceiving the humanness of older targets than younger ones because older targets are perceived as having higher experience.

Furthermore, brain functional connectivity between the two key brain regions for humanness perception, the ventral mPFC and left IFG, and other regions might coincide with stereotypes of older adults. Behavioral studies have reported that negative emotional reactions lead to dehumanizing perceptions (Buckels and Trapnell, 2013; Giner-Sorolla and Russell, 2019). Therefore, brain regions associated with negative emotions, such as the amygdala and insula (Phan et al., 2002), may be related to the activity of the ventral mPFC and the left IFG. Indeed, a previous MRI study showed that negative stereotype activation led to amygdala activation, which was also negatively correlated with activation in the prefrontal regions (Forbes et al., 2012). Therefore, if older adults are linked to negative stereotypes, especially along the agency dimension, there may be negative connectivity between key regions for humanness perception and emotion-related brain regions. According to previous studies, feelings of social connection are key factors in mind attribution (Epley et al., 2008; Saito et al., 2022). For example, people motivated to connect socially are more likely to attribute a higher experience of humanness toward others (Epley et al., 2008). Furthermore, people tend to attribute humanness to others when they feel socially accepted (Saito et al., 2022). Therefore, brain regions associated with social connectedness may correlate positively with the ventral mPFC and negatively with the left IFG. Previous neuroimaging studies have reported that the striatum and the ventral anterior cingulate cortex (vACC) are associated with social connectedness (Somerville et al., 2006; Gunther Moor et al., 2010; Hsu et al., 2020). For instance, the striatum and vACC are activated when people are socially accepted by others (Somerville et al., 2006; Hsu et al., 2020). The striatum is also activated when an individual expects to be accepted by a peer (Gunther Moor et al., 2010). Therefore, the striatum and/or vACC may have functional connectivity with the ventral mPFC and left IFG. Therefore, if older adults are linked to positive stereotypes, especially along the experience dimension (for example, evoking social connection), positive connectivity may appear between the key regions for humanness perception and social connection-related brain regions. Based on the abovementioned findings, we propose a third hypothesis regarding the association between brain connectivities and humanness perception:

We speculated that brain regions related to negative emotions, such as the amygdala and insula, and those related to social connectedness, such as the vACC and striatum, may show different functional connectivities with the key brain regions involved in humanness perception. This difference may be observed between older and younger targets.

As mentioned above, how the target’s age influences humanness perception and the underlying neural mechanisms, including functional connectivity among the brain regions involved in humanness perception, has remain unexplored. Functional connectivity analysis is important for understanding the fundamental organization of processing systems in the human brain (Krendl and Betzel, 2022). Thus, in this study, we investigated the effect of the target’s age on humanness perception and distinct neural activities by testing the three hypotheses mentioned above.

## 2 Methods

### 2.1 Participants

We determined the sample size for the current study based on the previous neuroimaging study (*n* = 27, Bruneau et al., 2018; *n* = 47, 40, Jack et al., 2013). Due to time constraints on data collection, we ultimately recruited 40 undergraduate and graduate students as participants. Written informed consent was obtained from each participant after the purpose and procedures of the study were explained. They received 3,000 JPY (about $26 USD) for their participation. The participants were recruited using a university bulletin board and a mailing list. All participants had normal or corrected-to-normal vision and no history of neurological or psychiatric illnesses. The data of five participants were excluded because of technical issues in which the MRI machine failed to collect complete brain data. Apart from this, there were no other instances of sample loss in the study. Finally, data from 35 participants (13 women, 22 men; mean_age_ = 20.54, SD_age_ = 1.63) were analyzed. A sensitivity power analysis for the within-participant *T* test, conducted in G*Power (Faul et al., 2007), indicated that, with our sample size, a minimum detectable effect size (d) was 0.487 using the following parameters: alpha = 0.05, power = 0.80. This study was approved by the Ethics Committee of the School of Medicine at Tohoku University and conducted in accordance with the Declaration of Helsinki.

### 2.2 Stimuli

We prepared 160 Japanese facial images collected from websites: 80 older faces (40 women and 40 men) and 80 younger faces (40 women and 40 men). All facial images were license-free and were viewed from the front. The images were converted to a 256 × 256 pixels grayscale image format with a white background. The images were rated by 9 independent participants (4 women, 5 men; mean_age_ = 22.11, SD_age_ = 1.05) in terms of attractiveness and the age of the faces. The attractiveness of the faces was assessed on a 4-point Likert scale ranging from 1 (less attractive) to 4 (highly attractive). Age perception was evaluated using an 8-point scale, with a range from 1 (ages 10–19) to 8 (ages 80–89). Based on these ratings, 80 images (40 older and 40 younger faces) were selected. Older faces were consistently perceived to be in their 60s to 80s (mean_rating_ = 6.57), while younger faces were perceived to be in their 10–20 s (mean_rating_ = 1.76). The sex ratios of the images were equal (women: men = 1:1). The mean attractiveness-rating scores for the selected 80 images were 2.67 (SD = 0.30) for older faces and 2.74 (SD = 0.23) for younger faces. Statistical analysis confirmed that the attractiveness scores of the groups did not significantly differ between the two groups [*t* (78) = 1.24, *p* = 0.22].

### 2.3 Apparatus

We programmed and conducted experimental tasks using PsychoPy version 1.85.2 (Peirce, 2007). All stimuli were presented on a 32” LCD monitor with an LED backlight intended to display visual stimuli for the fMRI experiments (BOLDscreen 32; Cambridge Research Systems, UK). Participants indicated their responses using two four-button response pads (HHSC-2 × 4-C; Current Designs Inc., Philadelphia, PA, United States).

### 2.4 Experimental task

The experimental task comprised two main tasks—involving agency and experience, respectively—and two control tasks, in which attractiveness and belonging were rated. We included control tasks, because these two factors have been reported to affect humanness perception. People are likely to attribute high levels of humanness to social connectedness (Haslam and Loughnan, 2014) and attractive targets (Alaei et al., 2022). Participants were provided with instructions prior to each task. The following instructions were used for the two main tasks: the agency task—“Please indicate the extent to which you feel the target has agency, which is the capacity to plan and act”; the experience task—“Please indicate the extent to which you feel a target has experience, which is the capacity to sense and feel pain and emotions such as pride.” For the two control tasks, the instructions were as follows: the attractiveness task—“Please indicate how attractive you find the target”; the belonging task—“Please indicate the extent to which you think the target would accept you.” The explanations for agency and experience were adapted from previous studies (Haslam, 2006; Gray et al., 2007). Although the previous studies used several questions and calculated two components, agency and experience, by averaging them (Haslam, 2006; Gray et al., 2007), we used only two questions about agency and experience owing to the time constraints on the MRI scanning time.

In each task, participants viewed 40 older and 40 younger faces and indicated their answers to each face based on the task instructions by pressing a button on a 6-point Likert scale ranging from 1 (not at all) to 6 (very much). The order of face presentation was randomized for each task. Before presenting faces, a fixation cross was presented as an interval jitter for 2 s (s), 4 s, or 6 s (frequency-weighted 2, 1, and 1, respectively).

### 2.5 Procedure

Before the experiment, the participants practiced responding to the task questions using two four-button response pads. The scanning session consisted of four runs. In each run, participants completed one task. Each run lasted approximately 10 min (min). After two runs (e.g., experience and agency tasks), the participants took a 20-min break outside the MRI scanner. The participants performed the remaining two tasks (e.g., attractiveness and belonging) in the third and fourth runs. After four runs, 3D T1-weighted images were assessed for approximately 10 min. The order of the tasks was counterbalanced across the participants.

### 2.6 Imaging parameter

All fMRI data were acquired using a 3 T Philips Achieva scanner (Philips Healthcare, Best, Netherlands) at the Institute of Development, Aging, and Cancer, Tohoku University. Functional images were acquired using a whole-brain continuous dual-echo sequence (64 axial slices; TR, 2,000 ms; TE, 12 and 35 ms; flip angle, 90°; slice matrix size, 64; slice sickness, 4 mm; and field of view [FOV], 240 mm). In each run, 304 volumes were obtained.

For each participant, a high-resolution 3D T1-weighted image was acquired with a magnetization-prepared rapid-acquisition gradient-echo (MPRAGE) sequence (162 axial slices; TR = 6.56 ms; TE = 3 ms; flip angle, 8°; slice matrix size, 240; slice thickness, 1 mm; FOV, 240 mm).

### 2.7 Behavioral data analysis

Analyses of behavioral data were conducted using R software (R Core Team, 2019). We examined the effect of the target age on perceived humanness ratings (agency and experience) using a generalized linear mixed model (GLMM). For the GLMM analysis, we used the lme4 package (Bates et al., 2015). The dependent variables were agency and experience ratings. The fixed effect was the target age (1 = older; 2 = younger). The random effects were the participants and face images. Additionally, attractiveness and belongingness ratings were included as covariates. Before analyzing data, we confirmed that the data followed a normal distribution by visually inspecting the data (see Supplementary Figure S1).

### 2.8 fMRI data analysis

The fMRI data were analyzed using Statistical Parametric Mapping 12 (SPM12; Welfare Department of Cognitive Neurology, London, UK), implemented in MATLAB 2017a.^1^ fMRI data preprocessing was performed according to the following procedure. First, functional images were realigned. Subsequently, slice-timing correction was applied to the images. Next, the images were co-registered to each participant’s MPRAGE image (T1 image) and spatially normalized to the Montreal Neurological Institute (MNI) template. Finally, the functional images were smoothed using a Gaussian kernel with an 8-mm full width at half maximum.

We used a multistage general linear model (GLM) to analyze the fMRI data. At the individual level, we estimated the trial-related brain responses separately for each run. Trials with no responses were omitted from the analysis. To examine the brain regions in which the response was linearly correlated with the rating scores for the agency, experience, attractiveness, and belonging tasks, the scores were entered into the model as first-order parametric modulators. In the experimental tasks, the bottom (1) and top (6) values were rarely used. Approximately 85% of the participants utilized these values in less than 5% of the trials. Furthermore, four participants did not use either the bottom or top values in their tasks. Therefore, we transformed the entered rating scores (1, 2, 3, and 4) from the original scores (1, 2, 3, 4, 5, and 6) to reduce the number of missing values. We grouped the original scores of 1 and 2 and those of 5 and 6; thus, the original scores were transformed from 1 and 2 to 1; 3 to 2; 4 to 3; and 5 and 6 to 4.

At the group level, we investigated the brain regions that correlate with humanness perception. For the analysis, we combined the parametric regressors of the two control conditions (attractiveness and belonging) as the baseline and subtracted them from those of experience or agency. In the analysis, the magnitude was modified such that the sum of the values in each contrast vector became zero; that is, [2 × experience-control conditions] and [2 × agency-control conditions]. Thus, we attempted to detect any brain area specifically corresponding to the degree of perceived humanness (experience or agency), controlling for the effect of the two parameters of no interest. We conducted these analyses for the older and younger target conditions. The statistical threshold for imaging results was set to *p* < 0.001, and the family wise error (FWE) was corrected for multiple comparisons at the cluster level *p* < 0.05.

Furthermore, we performed functional connectivity analysis using CONN toolbox version 18b.^2^ Given our *a priori* hypotheses, we set the left IFG and ventral mPFC as the seed regions. These regions were identified using the Harvard-Oxford Atlas in the CONN toolbox (Desikan et al., 2006). We specifically selected regions labeled as the Inferior Frontal Gyrus (pars triangularis left) for the left IFG and the Frontal Medial Cortex for the ventral mPFC. We used a statistical threshold of *p* < 0.05 FWE at the cluster level, with uncorrected *p* < 0.001 at the voxel level as the statistical threshold for connectivity analysis.

## 3 Results

### 3.1 Behavioral data analysis

Tables 1, 2 present the means and standard errors of the ratings and response times, respectively. We conducted GLMM analysis to assess the effect of the target age on the ratings of perceived humanness. We found a significant effect of target age on experience rating (*β* = −0.062, *z* = 2.350, *p* = 0.021; Supplementary Table S1). The standardized negative beta indicated that younger adults were attributed with a lower experience rating, indicating that older targets were perceived as having more experience than younger targets. However, there was no significant effect on agency rating (*β* = 0.007, *z* = 0.200, *p* = 0.842; Supplementary Table S2). The results of the behavioral data suggest that the target age influenced the two distinct dimensions of perceived humanness.

**Table 1:** Mean ratings of each condition.

	Old		Young		
Variable	*M*	SE	*M*	SE	*p*-value
Experience	3.516	0.128	3.496	0.112	0.021
Agency	3.446	0.116	3.631	0.088	0.842
Attractiveness	2.701	0.119	3.427	0.099	0.001
Belongingness	3.063	0.089	3.249	0.087	0.027

**Table 2:** Mean response time of each condition.

	Old		Young		
Variable	*M*	SE	*M*	SE	*p*-value
Experience	2.078	0.072	2.108	0.073	0.016
Agency	2.102	0.071	2.121	0.072	0.340
Attractiveness	1.982	0.073	2.018	0.072	0.073
Belongingness	2.092	0.073	2.157	0.077	0.004

We conducted GLMM analysis to assess the effect of target age on response times during the perceived humanness rating tasks. We found no significant effect on either experience or agency ratings (*βs* = 0.023, 0.014; *zs* = 1.404, 0.954; *ps* = 0.164, 0.340, respectively).

Furthermore, we conducted an explanatory analysis to assess whether the age of the participants influenced humanness perception toward both younger and older targets. The correlation analyses between humanness perception and participants’ ages showed that participants’ ages positively correlated with agency ratings toward older adults (Table 3).

**Table 3:** Correlations between humanness perceptions and participants’ age.

	Old		Young	
Variable	*r*	*p*-value	*r*	*p*-value
Experience	0.255	0.139	−0.285	0.098
Agency	0.520	0.001	−0.048	0.786

### 3.2 fMRI data: parametric modulation analysis

We collected 10,984 responses from all participants (2,829 responses for scores 1, 3,516 for scores 2, 2,678 for scores 3, and 1,961 for scores 4). There were no areas correlating with humanness perception (agency and experience) across comparisons between the humanness perception and control conditions. This result was inconsistent with our hypothesis, and we were unable to observe specific brain regions that correlated with the humanness ratings of older targets.

To examine the relationship between humanness ratings and the brain regions of interest (i.e., the ventral mPFC and left IFG), we conducted correlation analyses between the averaged beta values of the brain regions and humanness ratings as explanatory analyses. Although there were no significant correlations, we observed positive correlations between activities of the ventral mPFC and both agency and experience ratings (Supplementary Figure S2; *rs* = 0.212, 0.284, *ps* = 0.221, 0.098) and negative correlations between activities of the left IFG and both agency and experience ratings (*rs* = −0.205, −0.137, *ps* = 0.238, 0.433). These directions of correlations were consistent with our predictions.

### 3.3 fMRI data: functional connectivity analysis

**Figure 1:**
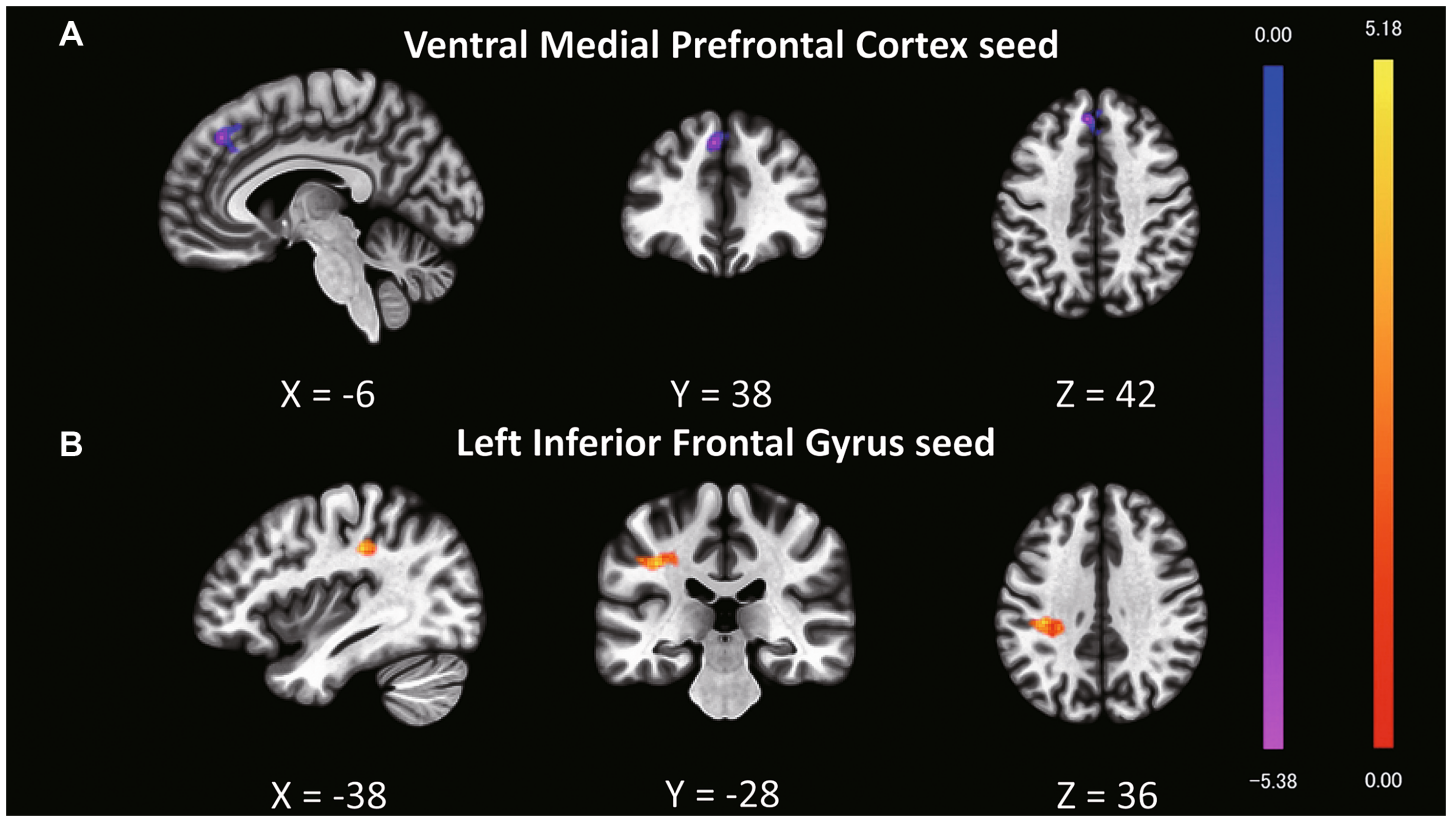
Clusters showing different functional connectivity levels with seed regions in a comparison of experience ratings for older and younger targets. The bright yellow regions represent greater values when older targets are perceived compared to younger targets. The bright purple regions represent weaker values when older targets are perceived compared to younger ones. **(A)** The ventral mPFC was set as the seed region, and a cluster including the dorsal mPFC, superior frontal gyrus (SFG), and dorsal anterior cingulate cortex (dACC) exhibited different connectivity patterns between older and younger targets. **(B)** The left IFG was set as the seed region, and a cluster including the supramarginal and postcentral gyri exhibited differential connectivity patterns between older and younger targets.

**Figure 2:**
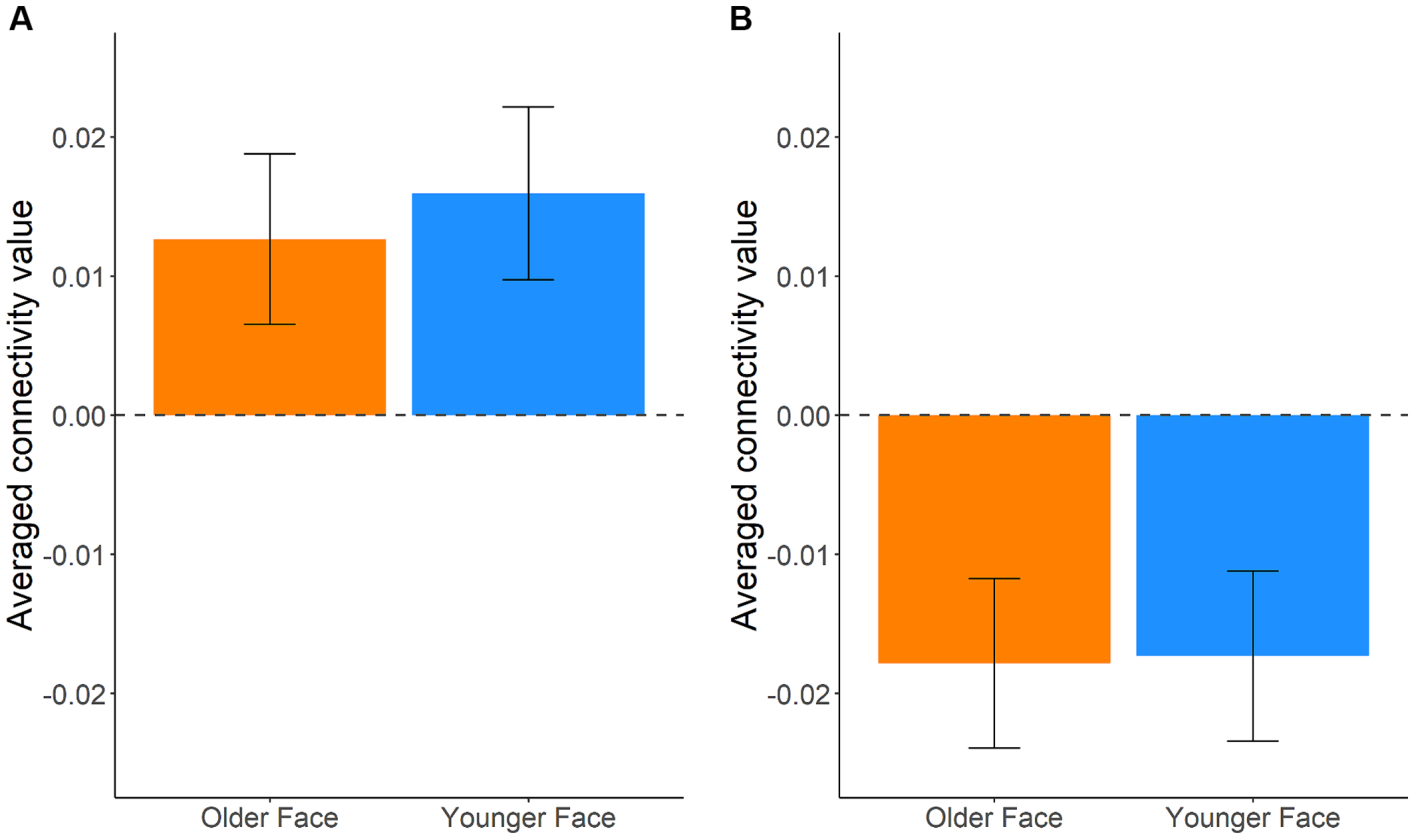
Average beta values between seed regions and clusters showing significant connectivity during experience rating. Error bars represent standard errors. **(A)** Average beta values of connectivity between the seed region (ventral mPFC) and a cluster that includes the dorsal mPFC, superior frontal gyrus (SFG), and dorsal anterior cingulate cortex (dACC). **(B)** Average beta values of connectivity between the seed region (left IFG) and a cluster that includes the postcentral and supramarginal gyri.

Although we found no significant main effect of task type and interaction, we analyzed the fMRI data based on the behavioral results. Behavioral data indicated that older targets were perceived as having more experience than younger targets; therefore, we expected to find a distinct functional connectivity underlying the different experience ratings of older and younger targets. To test this hypothesis, we performed functional connectivity analysis (seed-to-voxel analysis) with the ventral mPFC and left IFG as the seed regions (Table 4; Supplementary Table S3). Regarding the seed regions of the ventral mPFC, connectivity with a cluster including the dorsal mPFC, superior frontal gyrus (SFG), and dorsal anterior cingulate cortex (dACC) was significantly different between older and younger targets (Figure 1A; cluster size = 435, *p* = 0.002 FWE at the cluster level with uncorrected *p* < 0.001 at the voxel level, *t* = 5.51, peak voxel [−6, 38, 42]). Although the average connectivity values were positive for older and younger targets, the values were higher when the targets were younger compared to when they were older (Figure 2A). Regarding the seed region of the left IFG, connectivity with a cluster including the supramarginal gyrus and postcentral gyrus was significantly different between the older and younger targets (Figure 1B; cluster size = 295, *p* = 0.014 FWE at the cluster level with uncorrected *p* < 0.001 at the voxel level, *t* = 5.91, peak voxel [−38, −28, 36]). Although the average connectivity values for the ratings of older and younger targets were negative, the negative values were stronger when the targets were older compared to when they were younger (Figure 2B).

**Table 4:** Comparisons of functional connectivity for experience ratings of younger and older targets.

		Peak coordinates of the cluster					
Seed	Brain regions in the significant cluster	*x*	*y*	*z*	Size	*p*-value after cluster size FWE correction	*t*-value
vmPFC	dmPFC including SFG and dACC	−6	38	42	435	0.002	−5.51
left IFG	Postcentral gyrus and supramarginal gyrus	−38	−28	36	295	0.014	5.91

## 4 Discussion

### 4.1 Summary of findings

In this study, we used fMRI to investigate the effects of age on humanness perception in terms of agency and experience. We hypothesized that younger people would perceive older targets as having less agency and higher experience than younger targets and that the ventral mPFC and left IFG would parametrically correlate with agency and experience ratings. We found that the perception of experience was influenced by the target age. Inconsistent with a previous study in which younger people perceived older adults as having lower human ability (Boudjemadi et al., 2017), the current participants rated older targets as having more experience than younger targets. Additionally, inconsistent with our expectations, we did not find that the ventral mPFC and left IFG were correlated with humanness perception. In the subsequent analysis of functional connectivity during the experience rating, the connectivity between the ventral and dorsal mPFC was positively stronger when the targets were younger compared to when they were older. Furthermore, the connectivity between the left IFG and both the supramarginal and postcentral gyri was negatively stronger when the targets were older compared to when they were younger.

### 4.2 The effect of target age on perceived humanness

Consistent with our expectations, the participants rated the experience of older adults higher than that of younger adults. These results may reflect existing stereotypes that older people tend to be warm and tolerant (Fiske et al., 2002; Cuddy et al., 2005). However, these results are inconsistent with our hypothesis that older people’s agency would be perceived as lower than that of younger people. A previous study focusing on the dehumanization of older adults reported that they were animalistically dehumanized (Boudjemadi et al., 2017). Such dehumanization could be a failure to perceive the agency of a target, as individuals are linked to animals when they are perceived to be lacking uniquely human abilities (agency) such as self-control and rationality (Haslam and Loughnan, 2014). Thus, the results of this study appear inconsistent with prior research reporting animalistic dehumanization of older people (Boudjemadi et al., 2017). The experimental settings of the previous and present studies were quite different, which may explain why there was no significant difference in perceived agency between older and younger targets. Boudjemadi et al. (2017) used multiple approaches to assess the degree of animalistic dehumanization of older adults. In most of their experiments, participants were instructed to consider typical older people but not a specific older person and to respond with a dehumanizing attitude toward them. In contrast, in this study, participants judged the humanness of each older adult when viewing their faces individually. When people see another person as an individual rather than as part of a group, they tend to not link the person to a stereotype (Kunda and Sherman-Williams, 1993; Nelson, 2015) because information about an individual allows people to rely less on stereotypes. This may explain why participants in this study did not assign lower agency ratings to older adults.

### 4.3 Functional connectivity when judging the experience of older adults

We found that the strength of the functional connectivity between the ventral and dorsal mPFC, including SFG and the dACC, differed between older and younger targets when judging experience. Specifically, functional connectivity was stronger when the targets were younger compared to when they were older. Although it has been proposed that the dACC is critical for processing social rejection (Eisenberger et al., 2003), a recent study reported that the dACC is involved in the processing of self-relevant social evaluations, irrespective of valence (Perini et al., 2018). Therefore, the different connectivity levels between older and younger targets might reflect the involvement of self-relevant social evaluation in humanness perception, especially when the target’s age is similar to that of the perceiver. Considering that young targets are more self-relevant to participants because they are in the same age group, participants may have made self-relevant social evaluations, such as estimating whether they would be liked by the target. This evaluation may have led to the perception of the target as being more human-like, but only when the target was young.

The results of the behavioral data analysis indicated that older targets were attributed with having more experience, which was related to functional connectivity between the left IFG, left supramarginal gyrus, and left postcentral gyrus. Connectivity was negative and stronger in judgments for older targets than younger targets. Although this result was unexpected, it may indicate a relationship between humanness perception and emotional processing. Previous studies have revealed that the left postcentral gyrus is involved in the abstract representation of emotions (Baucom et al., 2012; Cao et al., 2018). Considering that it might be more difficult to estimate the emotional state of an older target than that of a young target, as older targets are in a different age group than young participants, unlike younger targets, emotional processing regarding the target might have been necessary when perceiving the humanness of older targets. Thus, the left postcentral gyrus may play a role in inhibiting the activation of the left IFG, leading to an increased experience rating when the target is older. Further studies are required for examining whether the aforementioned factors—self-relevance and emotional processing—mediate the perception of humanness toward younger and older targets.

### 4.4 Limitations and scope

The present study has some limitations. First, our primary purpose was to examine the effect of age on perceived humanness; however, we recruited only younger participants. The perception of humanness may be influenced by the perceiver’s age. Although the range of the participants’ ages was very limited, the correlation analysis showed that participants’ ages positively correlated with humanness perception toward older adults (i.e., agency ratings). Therefore, further studies that include both older and younger participants are necessary to consider the effects of age on the perception of humanness. Second, the rating moment overlapped with the picture presentation moment, making it difficult to distinguish between the neural patterns associated with image processing and those associated with ratings. Further studies must employ a more sophisticated procedure to identify brain activation related to humanness perception. Third, facial features were not considered. The morphological features of older and younger faces, such as eye slopes, differ (Chen et al., 2015). Thus, these age-related morphological changes in faces may have affected our results. Finally, we have not measured two dimensions of the stereotype content model: warmth and competence. We interpreted our behavioral results as reflecting warm and tolerant stereotypes of older adults. However, whether participants held those stereotypes toward older adults was unclear. Thus, further investigations are needed to investigate the relationship between stereotypes of age groups and humanness perception.

## Data availability statement

The raw data supporting the conclusions of this article will be made available by the authors, without undue reservation.

## Ethics statement

The studies involving humans were approved by the Ethics Committee of the School of Medicine at Tohoku University. The studies were conducted in accordance with the local legislation and institutional requirements. The participants provided their written informed consent to participate in this study.

## Author contributions

TS: Conceptualization, Data curation, Formal analysis, Funding acquisition, Investigation, Methodology, Project administration, Resources, Visualization, Writing – original draft, Writing – review & editing. RN: Conceptualization, Formal analysis, Methodology, Supervision, Validation, Writing – original draft, Writing – review & editing. RI: Data curation, Formal analysis, Methodology, Writing – original draft, Writing – review & editing. KM: Investigation, Writing – original draft, Writing – review & editing. YM: Investigation, Writing – original draft, Writing – review & editing. AK: Investigation, Writing – original draft, Writing – review & editing. MS: Methodology, Supervision, Writing – original draft, Writing – review & editing. RK: Resources, Supervision, Writing – original draft, Writing – review & editing.

## Footnotes

^1^ http://www.mathworks.com

^2^ http://www.nitrc.org/projects/conn
