# Supplementary material for: Perceiving humanness across ages: neural correlates and behavioral patterns
Source: Front Psychol. 2024 Apr 4;15:1361588. doi: 10.3389/fpsyg.2024.1361588 (PMC11024291; doi:10.3389/fpsyg.2024.1361588)
Supplement: Supplementary file 1 [file Data_Sheet_1.docx]

Supplementary Material

**Figure S1.** Histograms of behavioral data.


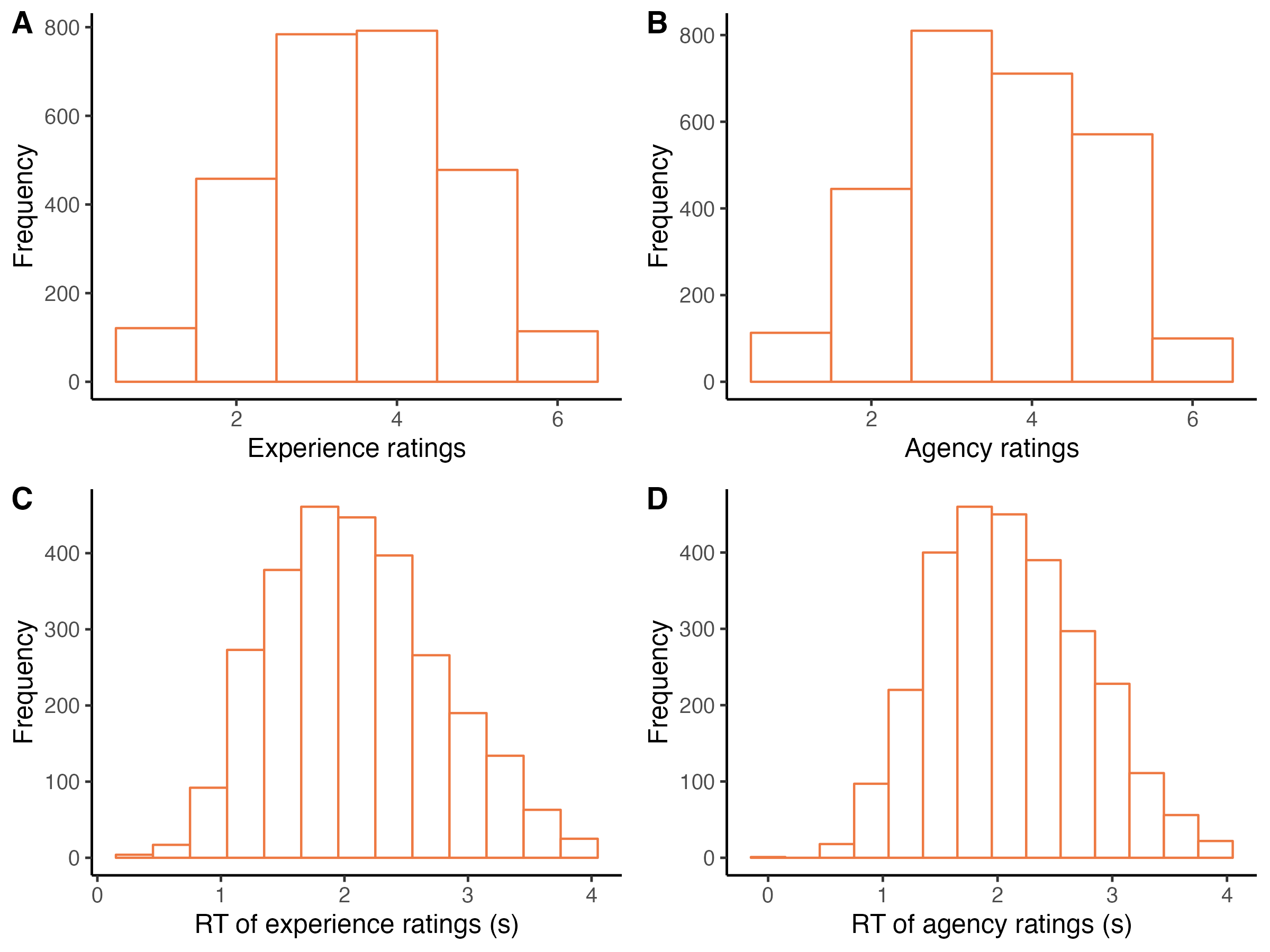


*Note.* Histograms of responses in (A) experience and (B) agency conditions. (C) Histograms of response time(s) in (C) experience and (D) agency conditions.

**Figure S2.** Correlations between brain region activities of interest and averaged humanness ratings.


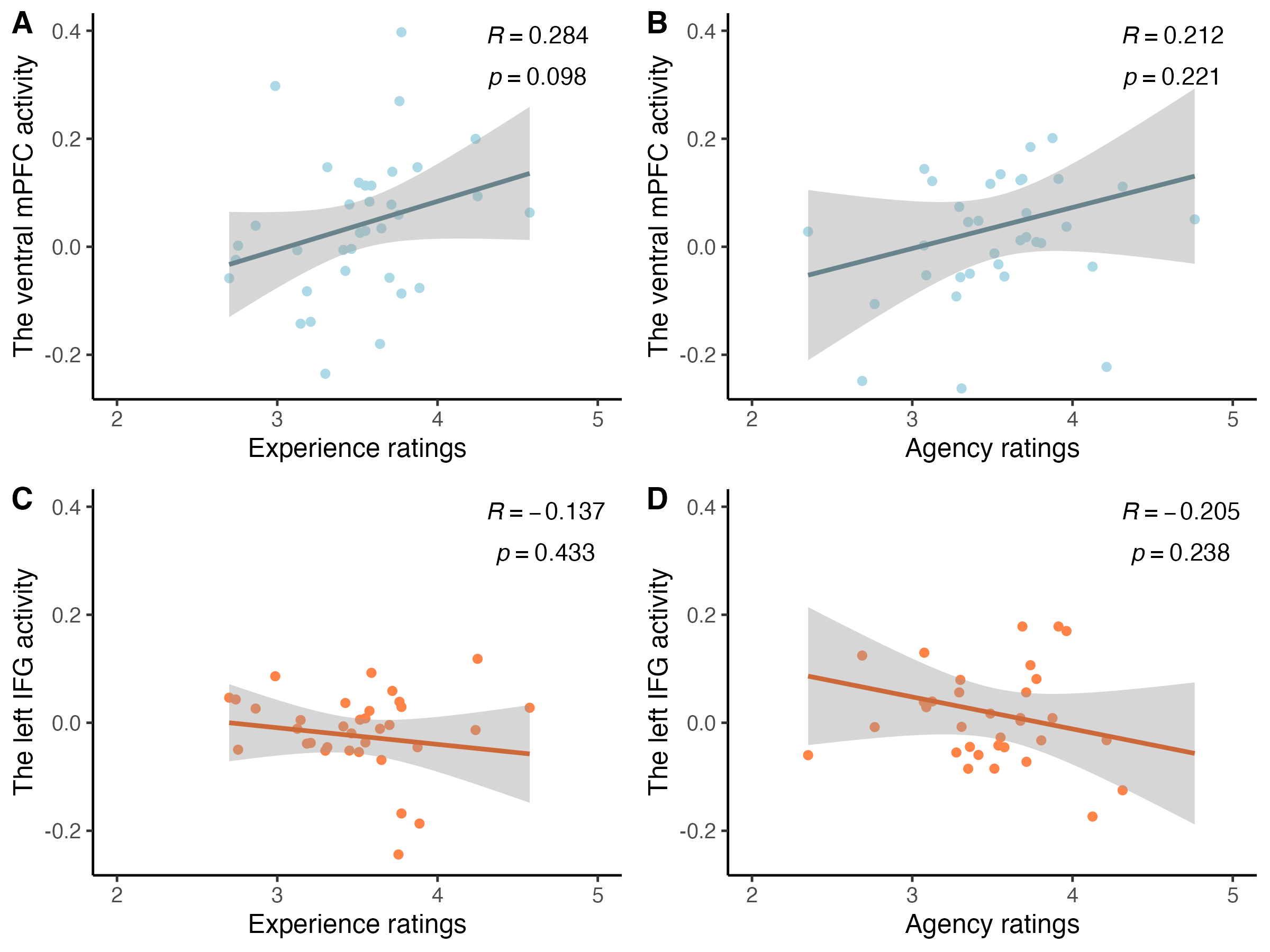


*Note.* Solid lines represent regression lines and the gray area represents 95% confidence intervals. Correlations between averaged beta values of the ventral mPFC and both (A) experience and (B) agency ratings. Correlations between averaged values of the left IFG and both (C) experience and (D) agency ratings.

**Table S1.** Generalized linear mixed model analysis of the effects of target age on experience rating.

| Random effects | SD | Variance |  |  |
| --- | --- | --- | --- | --- |
| Image | 0.202 | 0.041 |  |  |
| Participant | 0.124 | 0.124 |  |  |
|  |  |  |  |  |
| Fixed effects | Estimated *β* | SE | *z*-value | *p*-value |
| (Intercept) | 0.000 | 0.000 | 25.383 | < .001 |
| Target age | -0.062 | 0.063 | 2.350 | .021 |
|  |  |  |  |  |
| Covariates | Estimated *β* | SE | *z*-value | *p*-value |
| Belongingness | 0.184 | 0.021 | 8.975 | < .001 |
| Attractiveness | 0.139 | 0.022 | 6.333 | < .001 |

*Note.* Estimated β values were standardized.

**Table S2.** Generalized linear mixed model analysis of the effects of target age on agency rating.

| Random effects | SD | Variance |  |  |
| --- | --- | --- | --- | --- |
| Image | 0.360 | 0.129 |  |  |
| Participant | 0.425 | 0.181 |  |  |
|  |  |  |  |  |
| Fixed effects | Estimated *β* | SE | *z*-value | *p*-value |
| (Intercept) | 0.000 | 0.000 | 21.226 | < .001 |
| Target age | 0.007 | 0.063 | 0.200 | .841 |
|  |  |  |  |  |
| Covariates | Estimated *β* | SE | *z*-value | *p*-value |
| Belongingness | 0.144 | 0.037 | 7.406 | < .001 |
| Attractiveness | 0.196 | 0.019 | 9.375 | < .001 |

*Note*. Estimated β values were standardized.

**Table S3.** List of clusters showing different functional connectivity levels with seed regions in a comparison of experience ratings for older and younger targets.

| Seed | Brain areas | cluster size (voxels) |
| --- | --- | --- |
| vmPFC | Superior Frontal Gyrus Left | 133 |
|  | Superior Frontal Gyrus Right | 104 |
|  | Paracingulate Gyrus Left | 73 |
|  | Paracingulate Gyrus Right | 20 |
| left IFG | Postcentral Gyrus Left | 42 |
|  | Supramarginal Gyrus, anterior division Left | 40 |

*Note.* Labelling of brain areas is according to the Harvard-Oxford Atlas.
